# InCoB2014: bioinformatics to tackle the data to knowledge challenge

**DOI:** 10.1186/1471-2105-15-S16-I1

**Published:** 2014-12-08

**Authors:** Shoba Ranganathan, Tin Wee Tan, Christian Schönbach

**Affiliations:** 1 Department of Chemistry and Biomolecular Sciences and ARC Centre of Excellence in Bioinformatics, Macquarie University, Sydney NSW 2109, Australia; 2 Department of Biochemistry, Yong Loo Lin School of Medicine, National University of Singapore, Singapore 117599; 3 Department of Biology, School of Science and Technology, Nazarbayev University, Astana 010000, Republic of Kazakhstan; 4 Center for AIDS Research, Kumamoto University, Kumamoto 860-0811, Japan

## Abstract

Since 2006, the International Conference on Bioinformatics (InCoB) has been publishing selected papers in BMC Bioinformatics. Papers within the scope of the journal from the 13th InCoB July 31-2 August, 2014 in Sydney, Australia have been compiled in this supplement. These span protein and proteome informatics, structural bioinformatics, software development and bioimaging to pharmacoinformatics and disease informatics, representing the breadth of bioinformatics research in the Asia-Pacific.

## Introduction

InCoB (the International Conference on Bioinformatics) has served as the annual conference of the Asia-Pacific Bioinformatics Network (APBioNet) [1], since 2002 and Sydney, Australia was the venue for the 13^th^ InCoB, 31 July-2 August, 2014. In order to provide our region with international, peer-reviewed impact factor journal publications rather than printed books of conference proceedings, APBioNet has setup a rigorous peer review protocol and accepted the best InCoB papers in BMC Bioinformatics supplements since 2006, gradually, adding in BMC Genomics and BMC Systems Biology supplements, with BMC Medical Genomics as well this year. We have briefly reviewed the articles in this supplement, providing the 2014 bioinformatics research update from the APBioNet community.

## Manuscript submission and review

InCoB2014 provided authors the choice of submitting original research as full manuscripts to either the BMC track (supplement issues of BMC Bioinformatics, BMC Systems Biology or BMC Genomics) or to a special issue of PeerJ. The statistics for paper submission and acceptance, along with details of the peer review process undertaken as well as the links to the BMC Systems Biology, BMC Medical Genomics and PeerJ supplements are provided in the InCoB2014 BMC Genomics supplement introduction [2], with 16 “bioinformatics” articles briefly overviewed here.

## Protein and proteome informatics

Proteins display diverse functionality and these are usually the consequence of specific binding sites or sequence motifs. Dipeptide propensity scores have provided the solution to successfully predicting heme binding proteins [3]. Among biologically important post-translational modifications, O-linked glycosylation of serine and threonine residues is elusive in that there is no clear sequence motif associated with this site. Wu *et al.*[4] have applied support vector machine (SVM) learning to this problem, outperforming three other currently available tools. While mass spectrometry is frequently used to identify phosphorylated proteins, the low abundance of phosphopeptides in a sample is an obstacle to data analysis. iPhos [5] offers an innovative workflow system for streamlining phosphoproteome analysis.

## Structural bioinformatics

The biological function of a protein is ascribed to its 3D structure and five papers [6-10] provide updates on structural bioinformatics research. At the outset, for proteins that have neither experimental structural information nor structural homologues, predicting the 3D structure of a protein continues to remain a challenge. Paliwal *et al. *[6] have integrated evolutionary information and secondary structure prediction for protein 3D fold recognition, while Bhageerath-H [7] provides a novel *ab initio* and homology-based hybrid tertiary structure prediction server. For multi-domain proteins, the domain boundaries may be delineated by identifying inter-domain linker regions [8], for subsequent prediction of domain 3D structures using either [6] or [7]. Liu *et al. *[9] have analysed all available protein 3D structures and developed a new approach to discriminate between biologically relevant protein interactions and crystal packing contacts, while IFACEwat by Su *et al. *[10] seeks to predict near native structures of protein-protein complexes with the inclusion of solvent molecules at the interface.

## Bioinformatics software

Coevolution is an unusual phenomenon observed among species from different phyla asserting selective pressures on one another, making cophylogenetic analysis of these species very difficult. TreeCollapse addresses the cophylogeny problem indirectly by using common topological patterns [11]. In the area of bioimaging, phenotypic changes associated with development can be quantitatively analysed live imaging of muscle tissue using the software tool, FMAj [12]. These tools enable scientific advances in research areas where high quality software is lacking.

Where a number of bioinformatics software programs exist, quality assurance strategies are required to verify and validate the results generated. Ho and coworkers [13] show that metamorphic testing can be effectively applied to evaluate the results from two programs, BWA and Bowtie.

## Pharmacoinformatics and disease informatics

Generating a 3D map of chemical features of known ligands (a ‘pharmacophore’), for effective drug design remains a difficult problem which has been addressed by a pharmacophore-assisted iterative closest point (ICP) method [14]. Grover *et al*. [15] have used virtual screening of the glucagon receptor to identify novel natural inhibitors as potential therapeutic candidates for combating type 2 diabetes.

Prevention is better than cure, especially in the case of Alzheimer’s disease and Zhang *et al. *[16] propose a genetic algorithm with logistic regression for the early diagnosis of this disease from the information available from non-invasive neuropsychological tests. This could result in treatment options to prevent the onset or slow down the progression of this disease.

On the other hand, serious negative side reactions of drugs leading to organ failure can pose a serious threat to a subset of sensitive patients. To identify such patients prior to drug therapy, nephrotoxicity can be predicted based on only two genes [17] while multi-organ failure can be anticipated, using an integrative prediction score [18] for gene expression profiles.

## Conclusion

The articles in this supplement cover protein, proteome and structural bioinformatics, software packages as well as bioinformatics applications for drug development, early diagnosis of diseases and possible prevention of drug toxicity issues. We believe the Asia-Pacific is on track to participate in the ongoing NIH Big Data to Knowledge (DB2K) [19] and other similar global initiatives. We welcome you to attend our 2015 InCoB meeting to be held jointly with the Genome Informatics Workshop (GIW) in Tokyo, Japan [20], to contribute to this regional bioinformatics effort.

## Competing interests

None declared.

## Authors' contributions

SR wrote the introduction. CS and SR (Program Committee Co-chairs) managed the review and editorial processes, respectively. TWT supported the post-acceptance manuscript processing.

## Declarations

This article has been published as part of *BMC Bioinformatics* Volume 15 Supplement 16, 2014: Thirteenth International Conference on Bioinformatics (InCoB2014): Bioinformatics. The full contents of the supplement are available online at http://www.biomedcentral.com/bmcbioinformatics/supplements/15/S16.
